# Innsbruck REM Sleep Behavior Disorder Inventory may distinguish abnormal nocturnal movements related to obstructive sleep apnea

**DOI:** 10.1055/s-0045-1809543

**Published:** 2025-06-17

**Authors:** Gülçin Benbir Şenel, Ayşın Kısabay Ak, Ayşegül Şeyma Sarıtaş, Hikmet Yılmaz, Kübra Mehel Metin, Burcu Gökçe Çokal, Kadriye Ağan, Murat Aksu, Utku Oğan Akyıldız, Aylin Bican Demir, Betül Çevik, Ahmet Yusuf Ertürk, Derya Karadeniz, İbrahim Öztura, Gülin Sünter, Selma Tekin, İrsel Tezer, Deniz Tuncel Berktaş, Nazlı Totik, Kezban Aslan-Kara

**Affiliations:** 1 Istanbul University-Cerrahpasa, Cerrahpasa Faculty of Medicine, Department of Neurology, Division of Clinical Neurophysiology, Istanbul, Türkiye.; 2 Celal Bayar University, Medical Faculty, Department of Neurology, Manisa, Türkiye.; 3 University of Health Sciences, Ankara Training and Research Hospital, Department of Neurology, Ankara, Türkiye.; 4 Marmara University, Medical Faculty, Department of Neurology, Istanbul, Türkiye.; 5 Acibadem University, Atakent Hospital, Medical Faculty, Department of Neurology, Istanbul, Türkiye.; 6 Adnan Menderes University, Medical Faculty, Department of Neurology, Aydın, Türkiye.; 7 Bursa Uludag University, Medical Faculty, Department of Neurology, Bursa, Türkiye.; 8 Tokat Gaziosmanpasa University, Medical Faculty, Department of Neurology, Tokat, Türkiye.; 9 Dokuz Eylul University, Medical Faculty, Department of Neurology, Izmir, Türkiye.; 10 Pamukkale University, Medical Faculty, Department of Neurology, Denizli, Türkiye.; 11 Hacettepe University, Medical Faculty, Department of Neurology, Ankara, Türkiye.; 12 Kahramanmaras Sutcu Imam University, Medical Faculty, Department of Neurology, Kahramanmaras, Türkiye.; 13 Cukurova University, Medical Faculty, Department of Biostatistics, Adana, Türkiye.; 14 Cukurova University, Medical Faculty, Department of Neurology, Adana, Türkiye.

**Keywords:** REM Sleep Behavior Disorder, Sleep Apnea, Obstructive

## Abstract

**Background:**

Rapid eye movement (REM) sleep behavior disorder (RBD) is characterized by recurrent dream enactment behaviors like sleep-related vocalization and/or complex motor behaviors.

**Objective:**

To investigate the discriminative role of the validated Turkish version of the 9-Item Innsbruck REM Sleep Behavior Disorder Inventory (IRBD-9-Turkish) for idiopathic RBD (iRBD) in patients with obstructive sleep apnea (OSA).

**Methods:**

The current multicenter study was prospectively conducted in 13 accredited sleep centers in 10 different cities in Türkiye. Clinical data was obtained through a preformed questionnaire, and all participants were submitted to a full-night video-polysomnography (video-PSG) session in a sleep laboratory.

**Results:**

A total of 105 patients (mean age: 58.3 ± 11.6 years; 68.6% of male subjects) were prospectively and consecutively enrolled in the study; 51 patients (48.6%) presented iRBD, and 54 (51.4%), OSA, 19 (35.2%) of whom presented abnormal nocturnal behaviors (NBs) demonstrated by clinical and video-PSG findings associated with arousal reactions secondary to apneas and hypopneas. The cut-off value of the IRBD-9 was higher in patients with OSA-NBs than in those with OSA without NBs ( *p* < 0.001), with a sensitivity of 0.765 and a specificity of 0.667, resulting in a correct diagnosis of NBs in 75% of patients with OSA. The receiver operating characteristic (ROC) curves for Factor I (items 1, 2, 3, 6, and 8) and Factor II (items 4, 5, 7, and 9) of the IRBD-9-Turkish showed that both factors were able to distinguish patients with iRBD from those with OSA, but only Factor I distinguishes patients with iRBD from those with OSA-NBs.

**Conclusion:**

The present study demonstrated a very high sensitivity and specificity of the IRBD-9-Turkish not only in patients with iRBD, but also in patients with OSA.

## INTRODUCTION

Parasomnias are defined as undesirable verbal events, and/or motor movements, and/or experiences that emerge during or around sleep, which are classified into three groups depending on the sleep stage they arise as: non-rapid eye movement (NREM)-related parasomnias, rapid eye movement (REM)-related parasomnias, and other parasomnias (emerging irrespective of the sleep stage). 1

Rapid eye movement sleep behavior disorder (RBD) is one of the subtypes of REM-related parasomnias, which are characterized by recurrent dream enactment behaviors (DEBs) such as sleep-related vocalization and/or complex motor behaviors, which are documented or presumed to occur during REM sleep either through polysomnography (PSG) or clinical history. For a definitive RBD diagnosis, REM sleep without atonia (RSWA) should be demonstrated in PSG recordings. Lastly, the diagnosis of RBD can only be established if these disturbances are not better explained by another sleep disorder, mental disorder, or medication or substance use. 1 Idiopathic RBD (iRBD) and/or isolated RSWA is now accepted as one of the earliest prodromal biomarker of alpha-synucleinopathies, which comprise a set of neurodegenerative disorders including Parkinson's disease (PD), multiple system atrophy (MSA), and dementia with Lewy bodies (DLB). 2 3 4 Therefore, it is of crucial importance to differentiate iRBD/RSWA from secondary etiologies, associated conditions, and mimics.

The relationship between RBD and obstructive sleep apnea (OSA) is controversial, as some studies 5 suggest that the presence of RSWA in RBD may play as a protective role against OSA, while others 6 state that the presence of RBD may worsen OSA through anatomical interconnections in neural circuits of the lower brain stem and through alterations in neurotransmitters. On the other side, OSA contributes to the accumulation of abnormal misfolded proteins, alpha-synuclein, via sleep fragmentation and intermittent hypoxia, and results in acceleration of neurodegenerative diseases in the long term. 7 Moreover, arousal reactions associated with apneas and hypopneas in OSA may induce vivid dreaming and postarousal DEBs during REM sleep, which mimic RBD due to locomotion and agitated and violent behaviors. 8 9 These pseudo-RBD symptoms, termed *OSA-induced* , *hypnopompic* , *REM sleep-related parasomnias* , may represent a different type of confusional arousal that occurs in REM sleep following intense episodes of apneas and hypopneas associated with arousal reactions. The differential diagnosis of these attacks from isolated RBD requires a detailed evaluation and PSG investigation.

Parasomnias related to NREM sleep may also be easily misdiagnosed as iRBD. Especially in the pediatric age group, or in patients with narcolepsy, psychological distress and anxiety, DEBs may occur in NREM sleep rather than in REM sleep periods. 10 Alternative behaviors may also be confused with DEBs, as many sleepwalkers, or certain patients with sleep terrors have reported 11 vague contents of dreams with less elaborate content and less vivid experiences. Moreover, NREM and REM parasomnias may coexist, which is called *parasomnia overlap disorder* (POD). Overall, abnormal nocturnal behaviors (NBs) may result from different types of parasomnias, or they may originate from arousal reactions triggered by the apneas and hypopneas in OSA, or from periodic limb movements (PLMs), or present as part of other diseases such as nocturnal seizures or movement disorders. These similarities in clinical symptomatology and reciprocal interaction among sleep disorders, together with other mimicking conditions, emphasize the importance of a detailed and comprehensive clinical evaluation of these patients. There are questionnaires validated for screening for RBD, such as The Mayo Sleep Questionnaire, the RBD Screening Questionnaire (RBDSQ), the RBD Single Question Questionnaire (RBD-1Q), and the 9-Item Innsbruck REM Sleep Behavior Disorder Inventory (IRBD-9), but their usefulness is debatable. 12 13 Nevertheless, video-PSG for the definitive diagnosis of RBD is a time-consuming intensive method, and machine-learning techniques such as actigraphy require assisting with other clinical data, as in questionnaires. In the present study, we investigated the discriminative role of the validated Turkish version of the IRBD-9 (IRBD-9-Turkish) for iRBD regarding patients with OSA and those with OSA-related abnormal NBs (OSA-NBs).

## METHODS

### Sample selection

The present multicenter study was prospectively conducted in 10 different cities in Türkiye by the contributions of 13 sleep centers accredited by the Turkish Sleep Medicine Society (TSMS). Upon obtaining approval for the validation study for the IRBD-9-Turkish (developed by Frauscher et al. 13 ) and after the validation of the version, 14 we applied the IRBD-9-Turkish to our study sample. The current study was approved by the institutional Ethics Committee (MULA Reference: Turkish Sleep Study Group_TR_431114_MULA_20220608_FE), and the guidelines of the Strengthening the Reporting of Observational Studies in Epidemiology (STROBE) statement were followed. All participants provided written informed consent, and the study complied with the principles of the Declaration of Helsinki. The sample size was calculated based on statistical guidelines, 15 and the patients admitted to the outpatient sleep clinics of 13 different sleep centers in 10 different cities were prospectively and consecutively investigated to be enrolled into the study during the study protocol.

### Clinical and video-PSG analysis

The demographic and clinical history of the participants was recorded through a preformed questionnaire, and the presence of abnormal verbal and/or motor activities during sleep were specifically recorded in detail in all patients. Moreover, NREM parasomnias were interrogated in all participants.

All participants underwent full-night video-PSG recordings in accredited sleep laboratories that were recorded and scored by the sleep experts based on the most recent version of the American Academy of Sleep Medicine (AASM) Manual for the Scoring of Sleep and Associated Events. 16 A mandatory PSG characteristic of RBD, RSWA was accordingly scored in the electromyographic (EMG) recordings of chin and extremity electrodes (tibialis anterior muscle). The recording of EMG activity was performed in accordance with AASM criteria as follows: an amplification of 5 μV/mm, a low-frequency filter of 10 Hz, a high-frequency filter of 100 Hz, and a sampling frequency of 500 Hz. The presence of RSWA was defined as sustained (tonic) and phasic EMG activity scored from the chin and/or from the tibialis anterior muscle EMG channels. Tonic EMG activity was defined as an increase in the amplitude of the EMG electrode greater than the minimum amplitude demonstrated in NREM sleep and lasting for at least 50% of the duration of an epoch. Phasic EMG muscle activity was defined as bursts of transient muscle activity in at least 5 out of 10 (50%) 3-second mini-epochs, with each burst lasting for 0.1 to 5.0 seconds and at least 4 times as high in amplitude compared to the background EMG activity. 16 In addition, the total duration of phasic and tonic activity in the chin EMG channel and phasic activity in the extremity EMG channels were semiautomatically calculated for all REM sleep periods per night. Patients with a clinical history of repetitive complex motor movements and/or vocalization related to dream content during sleep, as well as those with proven RSWA in video-PSG recordings, were diagnosed as having RBD, based on the third edition (the most current version at the time of the study) of the International Classification of Sleep Disorders (ICSD-3). 17

Respiratory events were also recorded, scored, and analyzed in accordance with international guidelines, 16 and the apnea-hypopnea index (AHI) was calculated. The diagnosis of OSA was made based on the ICSD-3 17 criteria as follows: at least 1 relevant clinical symptomatology and AHI ≥ 5 events/hour, or AHI ≥ 15 events/hour with or without clinical symptomatology. Patients with OSA were divided into two groups, those with and without abnormal nocturnal movements, to be analyzed separately. The abnormal behaviors related to OSA are different from normal sleep behaviors (such as shifting body position or limb stretching, often described as “comfort movements”), but more complex behaviors like exploring the environment, defense behaviors, manipulative motor patterns or food-carrying behaviors. 18

The video-PSG parameters included the following: total recording time, total sleep time, sleep efficiency, sleep latency, REM sleep latency, wakefulness after sleep onset, the percentages of wakefulness and sleep stages (N1, N2, N3, and REM sleep), AHI, mean and minimum oxygen saturation, and the index of periodic limb movements in sleep ( **Supplementary Material** available at https://www.arquivosdeneuropsiquiatria.org/wp-content/uploads/2025/04/ANP-2024.0257-Supplementary-Material.docx - **Supplementary Material Table S1** ).

### Patient selection

After the detailed collection of clinical and PSG data, patients were enrolled into the study based on the inclusion and exclusion criteria. The inclusion criteria were volunteer patients aged ≥ 18 years, submitted to 1 night of video-PSG in a sleep laboratory accredited by the TSMS, with definitive diagnoses of iRBD and RSWA as demonstrated by video-PSG, and a definitive diagnosis of OSA.

On the other hand, patients with RBD secondary to narcolepsy or use of drugs such as antidepressant medications, other psychiatric and/or neurological conditions such as epilepsy and neurodegenerative disorders, deteriorated clinical condition such as severe medical condition or presenting cancer and/or alcohol or substance abuse were excluded.

The IRBD-9-Turkish was then applied to all participants, and the calculation of the item scores was carried out as it in the validation study. 14 The total RBD symptom score was calculated by dividing the total number of “yes” answers (that is, symptoms present) by the total number of questions. In the second section, the frequency scores of all items were added and divided by the total number of questions to obtain the RBD frequency score. The English and Turkish versions of the IRBD-9 are presented in **Supplementary Material Table S2** .

### Statistical analysis

Statistical analyses were performed using the IBM SPSS Statistics for Windows (IBM Corp.) software, version 20.0. Data were expressed as mean ± standard deviation or median and interquartile range (IQR) values. The Kruskal-Wallis test was used to compare more than two groups when the data was not normally distributed, and Pearson's correlation analysis was used to assess correlations between two normally-distributed variables. The reliability of the IRBD-9-Turkish was evaluated using the internal consistency (calculated through the Cronbach's alpha coefficient). The sensitivity and specificity of various cut-off values were determined and shown using a receiver operating characteristic (ROC) curve. The Cronbach's alpha coefficient was determined to assess the questionnaire's reliability. An area under curve (AUC) greater than 0.70 was deemed sufficient, and a Cronbach's alpha coefficient greater than 0.7 was regarded as satisfactory. 19 The scale's structural validity was evaluated using exploratory factor analysis (EFA). Construct validity of the IRBD-9-Turkish was investigated through EFA (as described in detail in the validation study 14 ), which showed a 2-factor solution for the IRBD-9-Turkish with the rotated factor loadings ranging from 0.61 to 0.92. Therefore, the 2-factor model explained a significant portion of the scale variation: five items (questions 1, 2, 3, 6, and 8) were loaded on factor I, and the other items (questions 4, 5, 7, and 9) were loaded on factor II. Values of *p* ≤ 0.05 were considered statistically significant.

## RESULTS

### Study population

Current study involved 105 participants with a mean age of 58.3 ± 11.6 years and 68.6% of males subjects. The mean symptom score on the IRBD-9-Turkish of the total sample was of 0.36 ± 0.25 points. In total, 51 patients presented iRBD, and 54, OSA. The mean symptom score was significantly higher in iRBD patients compared with OSA patients ( Table 1 ).

**Table 1:** Comparison of the descriptive statistics of iRBD and OSA patients

		Patients with iRBD (n = 51)	Patients with OSA (n = 54)	*p* value
Sex: n (%)	Male (n = 131)	32 (62.7)	40 (74.1)	0.299
	Female (n = 107)	19 (37.3)	14 (25.9)	
Mean age (years)		64.90 ± 7.69	51.96 ± 11.19	< 0.001
Mean IRBD-9-Turkish total score (symptom)		0.56 ± 0.20	0.17 ± 0.13	< 0.001
Mean RBDSQ-T total score		8.67 ± 1.87	2.69 ± 2.45	<0.001
Mean RBD duration (months)		50.18 ± 69.90	NA	–
Mean PLMSI		15.97 ± 20.36	10.35 ± 16.21	0.123
RSWA fulfilling the AASM criteria: n (%)	None	0 (0.0)	53 (98.1)	< 0.001
	Chin alone	0 (0.0)	0 (0.0)	
	Extremity alone	2 (3.9)	1 (1.9)	
	Chin and extremity	49 (96.1)	0 (0.0)	
Mean duration of increased phasic/tonic activity in the chin EMG channel		156.26 ± 482.56	0.15 ± 0.31	< 0.001
Mean duration of increased phasic activity in the extremity EMG channels		61.58 ± 220.90	0.18 ± 0.51	< 0.001

Abbreviations: AASM, American Academy of Sleep Medicine; EMG, electromyography; iRBD, idiopathic rapid eye movement sleep behavior disorder; IRBD-9-Turkish, Turkish version of the 9-item Innsbruck Rapid Eye Movement Sleep Behavior Disorder Inventory; NA, not applicable; OSA, obstructive sleep apnea; PMLSI, Periodic Limb Movements in Sleep Index; RBDSQ-T, Turkish Version of the Rapid Eye Movement Sleep Behavior Disorder Questionnaire; RSWA, rapid eye movement sleep without atonia.

### Validation of the IRBD-9-Turkish inventory

Construct validity analysis for the symptom score section showed a 2-factor model for the IRBD-9-Turkish, with questions 1, 2, 3, 6, and 8 loaded onto factor I, and questions 4, 5, 7, and 9 loaded onto factor II (as described in the validation study 14 ). The mean scores on factors I and II showed that iRBD patients scored significantly higher on factor I than the OSA patients ( Table 2 ). The mean scores on factor II were also higher in iRBD patients, which was not statistically significant.

**Table 2:** Comparison of the mean scores on factors I and II of the exploratory factor analysis between iRBD and OSA patients

Mean symptom score	Patients with iRBD (n = 51)	Patients with OSA (n = 54)	*p* -value
Factor I (questions 1,2,3,6,8 of the IRBD-9-Turkish)	0.78 ± 0.21	0.13 ± 0.17	< 0.001
Factor II (questions 4,5,7,9 of the IRBD-9-Turkish)	0.29 ± 0.28	0.23 ± 0.18	0.522

Abbreviations: iRBD, idiopathic rapid eye movement sleep behavior disorder; IRBD-9-Turkish, Turkish version of the 9-item Innsbruck Rapid Eye Movement Sleep Behavior Disorder Inventory; OSA, obstructive sleep apnea.

Criterion validity analysis showed that the optimal cut-off value of the IRBD-9-Turkish was of 0.28 for healthy individuals compared with the iRBD patients, with a sensitivity of 0.941 and a specificity of 0.947, resulting in correct diagnosis in 94.4% of the patients (as shown in the validation study 14 ). Patients with OSA also displayed a significantly lower cut-off value, of 0.278, with a sensitivity of 0.941 and a specificity of 0.889, resulting in correct diagnosis in 91.2% of the patients ( Table 3 ).

**Table 3:** Diagnostic performance of the IRBD-9-Turkish symptom section in OSA patients compared to iRBD patients

**Diagnostic performance of the IRBD-9-Turkish inventory symptom section**									
**In comparison to iRBD**	Cut-off	Sensitivity (%)	Specificity (%)	PPV (%)	NPV (%)	LR (+)	LR (-)	AUC	*p* value
**OSA**	0.278	94.1	88.9	88.9	94.1	8.47	0.07	0.95	< 0.001
**Diagnostic performance of the IRBD-9-Turkish inventory symptom section for factors I and II**									
**Patients with iRBD and OSA**	Cut-off	Sensitivity (%)	Specificity (%)	PPV (%)	NPV (%)	LR (+)	LR (-)	AUC	*p* value
**Factor I**	0.300	96.1	90.7	90.7	96.1	10.4	0.04	0.973	< 0.001
**Factor II**	0.375	29.4	88.9	71.4	57.1	2.65	0.79	0.533	0.557

Abbreviations: AUC, area under the curve; iRBD, idiopathic rapid eye movement sleep behavior disorder; IRBD-9-Turkish, Turkish version of the 9-item Innsbruck Rapid Eye Movement Sleep Behavior Disorder Inventory; LR(+), positive likelihood ratio; LR(-), negative likelihood ratio; NPV, negative predictive value; OSA, obstructive sleep apnea; PPV, positive predictive value.

### IRBD-9-Turkish inventory in patients with OSA and OSA-NB

**Figure 1:**
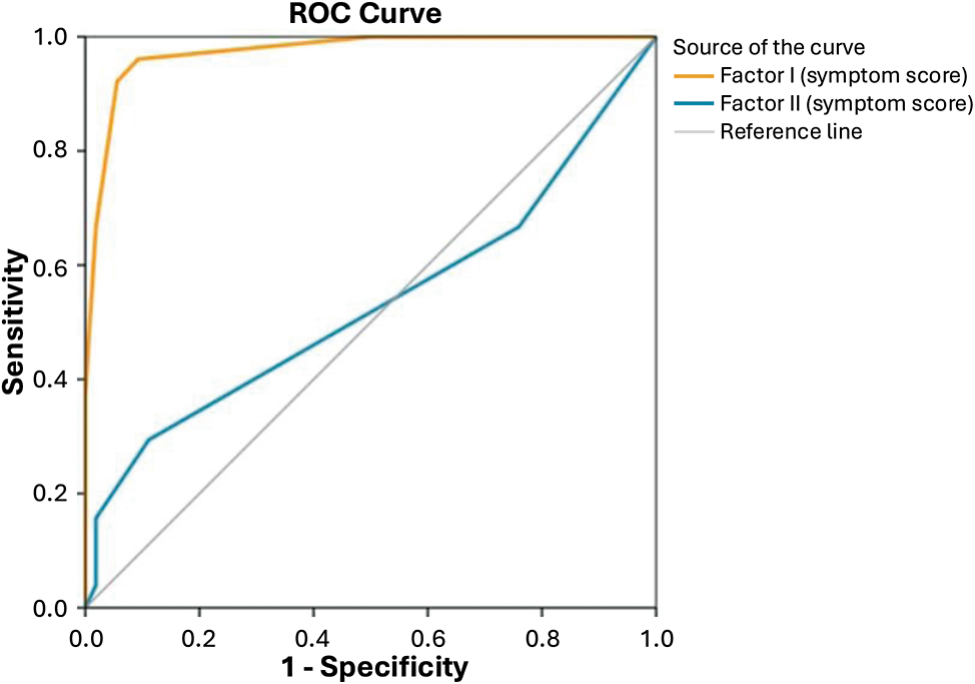
Graph illustrating the receiver operating characteristic (ROC) curves: area under the curve (AUC) for factor I = 0.973 (95%CI: 0.946–1.000; *p* < 0.001); and for factor II = 0.533 (95%CI: 0.419–0.647; *p* = 0.557).

The diagnostic performance of the symptom section of the IRBD-9-Turkish was also observed to be significant for factor I, but not for factor II ( Table 3 ). The ROC curves were able to separate patients with iRBD from patients with OSA through an analysis of factors I and II ( Figure 1 ). Of the 54 OSA patients, 19 (35.2%) presented OSA-NB, demonstrated by both clinical and video-PSG findings, which were associated with the arousal reactions secondary to apneas and hypopneas; while 39 patients (64.8%) had no symptoms nor signs of NBs. Descriptive statistics of these two groups are shown in Table 4 .

**Table 4:** Descriptive statistics of OSA and OSA-NBs patients

		Patients with OSA-NBs (n = 19)	Patients with OSA (n = 39)	*p* value
Sex: n (%)	Male (n = 40)	11 (61.1)	29 (74.4)	0.326
	Female (n = 17)	7 (38.9)	10 (25.6)	
Mean age (years)		50.5 ± 11.01	52.56 ± 10.92	0.264
Mean IRBD-9-Turkish total score (symptom)		0.35 ± 0.19	0.13 ± 0.08	< 0.001
Mean RBDSQ-T total score		4.39 ± 2.83	2.28 ± 2.32	0.001
Mean duration of increased phasic/tonic activity in chin EMG channel		0.06 ± 0.24	0.18 ± 0.34	< 0.001
Mean duration of increased phasic activity in extremity EMG channels		0.07 ± 0.31	0.22 ± 0.57	< 0.001

Abbreviations: EMG, electromyography; IRBD-9-Turkish, Turkish version of the 9-item Innsbruck Rapid Eye Movement Sleep Behavior Disorder Inventory; NBs, abnormal nocturnal behaviors; OSA, obstructive sleep apnea; RBDSQ-T, Turkish Version of the Rapid Eye Movement Sleep Behavior Disorder Questionnaire.

**Figure 2:**
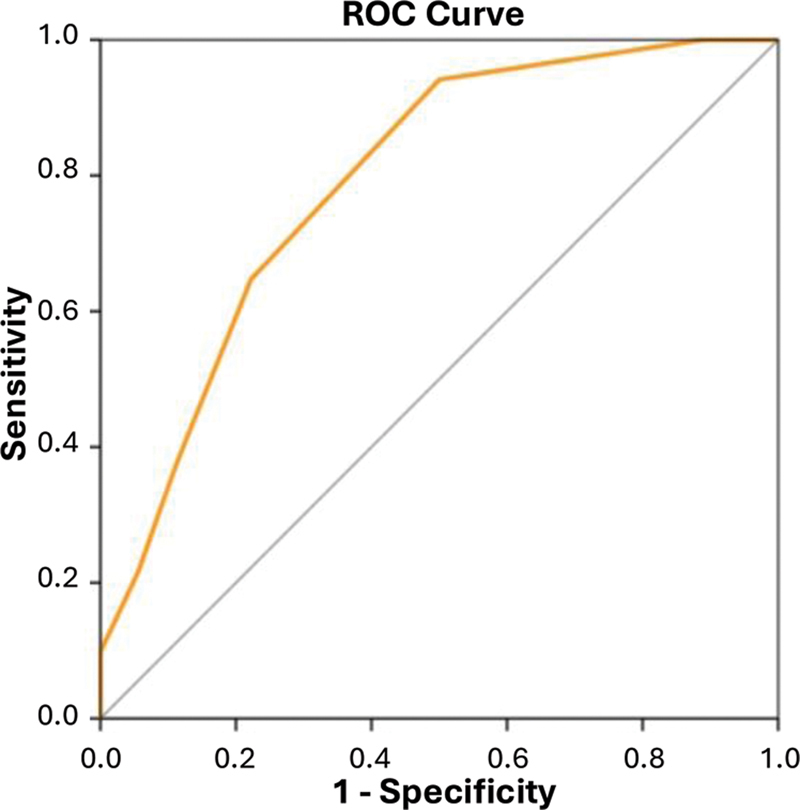
Graph showing the ROC curve using the symptom score of the Turkish version of the 9-Item Innsbruck REM Sleep Behavior Disorder Inventory (IRBD-9-Turkish) to distinguish patients with idiopathic rapid eye movement sleep behavior disorder (iRBD) from the those with obstructive sleep apnea-related abnormal nocturnal behaviors (OSA-NBs) (AUC = 0.791; 95%CI: 0.661–0.921; *p* < 0.001).

The analysis of the subgroups of patients with OSA and OSA-NBs through a stratified analysis of the symptom section of the IRBD-9-Turkish revealed that the cut-off value was significantly higher in patients with OSA-NBs (0.39 versus 0.278 for those with OSA; *p* < 0.001), with a sensitivity of 0.765 and a specificity of 0.667, resulting in a correct diagnosis of BNs in 75% of the patients with OSA ( Table 5 ). The ROC curve using the IRBD-9-Turkish symptom score was able to distinguish patients with iRBD from patients with OSA-NBs ( Figure 2 ).

**Table 5:** Group-by-group stratified analysis of the symptom section of the IRBD-9-Turkish inventory

In comparison to RBD	Cut-off	Sensitivity (%)	Specificity (%)	PPV (%)	NPV (%)	LR (+)	LR (-)	AUC	*p* -value
OSA	0.28	94.1	100.0	100.0	92.9	–	0.06	0.99	< 0.001
OSA-NB	0.39	76.5	66.7	86.7	50.0	2.29	0.35	0.79	< 0.001

Abbreviations: AUC, area under the curve; IRBD-9-Turkish, Turkish version of the 9-item Innsbruck Rapid Eye Movement Sleep Behavior Disorder Inventory; LR(+), positive likelihood ratio; LR(-), negative likelihood ratio; NBs, abnormal nocturnal behaviors; NPV, negative predictive value; OSA, obstructive sleep apnea; PPV, positive predictive value; RBD, rapid eye movement sleep behavior disorder.

**Figure 3:**
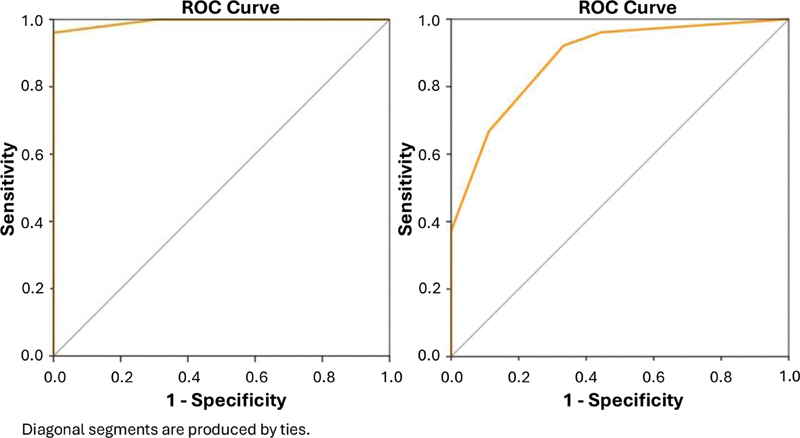
Graph showing the ROC curve using the IRBD-9-Turkish symptom score for factor I, which distinguishes iRBD from OSA patients (AUC = 0.994; 95%CI: 0.983–1.000; *p* < 0.001); sensitivity = 0.961; specificity = 1.000; cut-off = 0.30) ( **A** ) and iRBD patients from those with OSA-NBs (AUC= 0.883; 95%CI: 0.797–0.970; *p* < 0.001); sensitivity = 0.922; specificity = 0.667; cut-off = 0.50).

The analysis of ROC curves for factors I and II showed that the IRBD-9-Turkish symptom score was able to distinguish patients with iRBD from patients with OSA ( Figure 3A ), and OSA-NBs ( Figure 3B ) only regarding factor I, but not factor II.

## DISCUSSION

The current study demonstrated a very high sensitivity and specificity of the IRBD-9-Turkish in iRDB and OSA patients. The diagnostic performance, however, was observed to be significant for factor I, but not for factor II. More intriguingly, our findings showed that the IRBD-9-Turkish was also able to distinguish patients with iRBD from patients with OSA-NBs in the factor I model. The cut-off value for the IRBD-9-Turkish inventory was observed to be significantly higher for OSA-NBs patients (0.39) compared to those with OSA (0.278) or iRBD (0.28). With a high sensitivity and specificity, the IRBD-9-Turkish predicted the correct diagnosis of BNs in 75% of the OSA patients.

The IRBD-9 was developed and validated with a cutoff score of 0.25, showing an excellent sensitivity and speciﬁcity (of 0.914 and 0.857 respectively). 13 Although it has been shown to differentiate patients with RBD from those without it regardless of underlying etiology, no difference in scores were observed between patients with idiopathic versus symptomatic RBD. On the other hand, BNs triggered by apneas and hypopneas in OSA are very common and challenging in the differential diagnosis of RBD in clinical practice. Considering that video-PSG is a time-consuming and intensive method, the use of questionnaires to choose and prioritize patients who will undergo later or and earlier investigations like such as and PSG would be very helpful. 20 To the best of our knowledge, no questionnaire has been developed for the assessment of OSA-NBs. Our results, noticeably, showed that the The IRBD-9 can also be used to distinguish OSA-NBs from RBD-DEBS. While the definitive diagnosis of RBD requires a detailed evaluation and video-PSG recordings, the use of the IRBD-9 as a screening method in clinical practice, as well as in epidemiological population-based settings, must be considered.

Indeed, there are some other instruments validated for the assessment of nocturnal behaviors other than RBD. Spoormaker et al. 21 developed the SLEEP-50 questionnaire, which was designed to assess many sleep complaints and/or disorders, such as apneas, insomnia, narcolepsy, sleep paralysis, restless legs syndrome, sleep-related cramps, circadian rhythm sleep disorders, nightmares, and sleepwalking, aiming to distinguish sleep complaints from sleep disorders. The Global Sleep Assessment Questionnaire (GSAQ), developed by Roth et al. 22 to recognize many sleep disorders, including PLMs and parasomnias. Fulda et al. 23 developed the Munich Parasomnia Screening (MUPS) to evaluate the occurrence and the frequency of different groups of patients with nocturnal behaviors, such as sleep-related movement disorders, isolated symptoms and normal variants, and parasomnias. There are also other questionnaires that screen for more than one sleep disorder at the same time, 24 but, in all, the questionnaires are focused on a specific sleep disorder, and OSA-NBs has not been contemplated in any of them. The evaluation of iRBD through actigraphy or actigraphy and screening questionnaires was also analyzed in a study 20 that showed that actigraphy recordings for 7 to 10 nights were able to detect iRBD, with a high sensitivity (95.2%) and specificity (90.5%), while the simultaneous use of actigraphy and questionnaires presented 100% of precision, with a sensitivity of 88.1%. Statistical models incorporating clinical features (loss of smell, autonomic dysfunction etc) and/or neurophysiological markers (such as a decrease in fast-frequency activities in spectral electroencephalography) may improve the clinical diagnostic procedure in iRBD patients. 20 25

The current study has strengths and limitations. Our results demonstrated the use of the IRBD-9y in a new field of sleep medicine: OSA-NBs. The sensitivity and specificity of the IRBD-9-TR were very high to differentiate patients with OSA from those with iRBD, as well as those with OSA-NBs, a condition that can mimic isolated RBD. On the other hand, the study sample was small. In addition, NBs associated with apneas and hypopneas were not further grouped into those arising during NREM sleep and those arising during REM sleep.

Despite its limitations, the present study also has strengths. It is a prospective multicenter study representative of sleep centers in tertiary neurology clinics. The IRBD-9-TR was applied to subjects referred to the sleep laboratory for the first time. All participants were able to fill it out by themselves, without the help of the clinicians, which shows the user-friendly character of the questionnaire for screening purposes. Considering that, video-PSG investigations are time-consuming and expensive procedures, and the use of RBD screening questionnaires would be very useful for clinicians and researchers. While the IRBD-9 was designed for the differentiation of RBD from other sleep disorders, its discriminative role regarding patients with OSA and OSA-NBs was supported and strengthened by the results of the current study. This additional characteristic might further help the differential diagnosis of iRBD from its mimics, which can be very challenging in the daily clinical practice. Nevertheless, the results of the present study warrant the conduction of larger studies with a more detailed analysis of OSA-NBs. On the other hand, the important ethical implications of the diagnosis of iRBD as a prodromal feature of alpha-synucleinopathy 3 4 should emphasize the role of screening tests as an intermediate step in choosing and prioritizing patients for further evaluations, including video-PSG.

**Table S1:** Polysomnographic data of the study sample

Parameters	Patients with iRBD (n = 51)	Patients with OSA (n = 54)	*p* -value
Mean total recording time (minutes)	433.0 ± 57.3	442.0 ± 46.0	0.789
Mean total sleep time (minutes)	361.7 ± 76.7	384.6 ± 62.2	0.250
Mean sleep efficiency (%)	79.8 ± 13.7	81.8 ± 20.2	0.075
Mean sleep latency (minutes)	24.5 ± 23.3	24.9 ± 30.9	0.353
Mean REM sleep latency (minutes)	129.2 ± 72.3	148.8 ± 92.1	0.436
Mean wakefulness after sleep onset (minutes)	56.0 ± 38.7	55.0 ± 51.5	0.454
Mean N1 sleep (%)	8.4 ± 6.3	12.7 ± 11.5	0.031
Mean N2 sleep (%)	44.6 ± 15.0	54.6 ± 12.5	0.001
Mean N3 sleep (%)	25.2 ± 11.0	16.1 ± 10.4	0.001
Mean REM sleep (%)	20.0 ± 9.4	15.3 ± 8.3	0.014
Mean apnea-hypopnea index (events per hour)	5.6 ± 4.8	46.1 ± 19.8	< 0.001
Mean minimum oxygen saturation (%)	93.8 ± 2.1	89.6 ± 11.1	< 0.001
Mean Index of Periodic Limb Movements in Sleep (per hour)	9.7 ± 18.7	11.0 ± 16.6	0.580

**Abbreviations:** iRBD, idiopathic rapid eye movement sleep behavior disorder; REM, rapid eye movement; OSA, obstructive sleep apnea.

**Table S2:** Original English version and Turkish version of the 9-item Innsbruck Rapid Eye Movement Sleep Behavior Disorder Inventory (IRBD-9)

**ENGLISH** 1. Do you dream of violent or aggressive situations (e.g., to have to defend yourself)? 2. Do you scream, insult, or curse during your sleep? (Note: this does not include normal sleep talking.) 3. Do you move out of your sleep and occasionally perform ‘‘flailing’' or more extensive movements? 4. Have you left your bed and have you gone out of the room during your sleep since entering your adulthood? 5. Have you ever fallen out of bed while you were sleeping? 6. Have you ever injured or nearly injured yourself or your bed partner while you were sleeping? 7. Have you ever found items that were placed on the bed table when falling asleep lying on the floor when you awakened (eg, alarm clock, mobile phone, etc.)? 8. Are the above-described movements out of your sleep occasionally or always in line with the content of your dreams? (items 2, 3, 6) 9. Do you snore loudly and irregularly, or do you know you have an irregular breathing during your sleep? **TURKISH** 1. Uykunuzda, şiddet veya saldırganlık içeren rüyalar görür müsünüz? (kendinizi korumak zorunda kaldığınız rüyalar ve benzeri) 2. Uykunuzda çığlık atıp, hakaret ediyor veya küfür ediyor musunuz? (Not: normal uyku konuşması dâhil değildir) 3. Uykunuzda hareket ediyor ve ara sıra “sıçrama-silkelenme” veya daha aşırı hareketler yapıyor musunuz? 4. Erişkin olduğunuzdan beri, uykunuzda yatağınızı terk edip odadan çıktığınız oldu mu? 5. Uyurken hiç yataktan düştünüz mü? 6. Uyurken kendinizi veya eşinizi yaraladınız mı? veya neredeyse yaralayacak oldunuz mu? (örn: istem dışı vurdunuz mu?) 7. Uykuya dalarken komodinin üzerinde olan eşyaları uyandığınızda yerde bulduğunuz oldu mu? (örneğin çalar saat, cep telefonu vb.) 8. Yukarıda tanımlanan uykudaki hareketler, ara sıra veya sürekli olarak gördüğünüz rüyalarınızın içeriği ile aynı mı? (2, 3, 6 maddeler) 9. Yüksek sesle ve düzensiz bir şekilde horlar mısınız veya uyurken düzensiz nefes aldığınızı biliyor musunuz?
